# Prevalence of blood and skin trypanosomes in domestic and wild fauna from two sleeping sickness foci in Southern Cameroon

**DOI:** 10.1371/journal.pntd.0011528

**Published:** 2023-07-27

**Authors:** Eugenie Melaine Kemta Magang, Rolin Mitterran Ndefo Kamga, Jenny Telleria, Magali Tichit, Aline Crouzols, Jacques Kaboré, David Hardy, Calmes Ursain Tsakeng Bouaka, Vincent Jamonneau, Brice Rotureau, Victor Kuete, Jean-Mathieu Bart, Gustave Simo

**Affiliations:** 1 Molecular Parasitology & Entomology Sub-unit, Department of Biochemistry, Faculty of Science, University of Dschang, Dschang, Cameroon; 2 Univ. Montpellier, CIRAD, IRD, Intertryp, Montpellier, France; 3 Histopathology Platform, Institut Pasteur, Paris, France; 4 Trypanosome Transmission Group, Trypanosome Cell Biology Unit, INSERM U1201, Department of Parasites and Insect Vectors, Institut Pasteur Paris, Université Paris Cité, Paris, France; 5 Centre International de Recherche-Développement sur l’Elevage en zone Subhumide, Unité de recherche sur les maladies à vecteurs et biodiversité, Bobo-Dioulasso, Burkina Faso; 6 Centre for research in infectious disease, Yaoundé, Cameroon; 7 Unité de Recherche « Trypanosomoses », Institut Pierre Richet, Bouaké, Côte d’Ivoire; 8 Parasitology Unit, Institut Pasteur of Guinea, Conakry, Guinea; 9 Research Unit of Microbiology and Antimicrobial Substances, Department of Biochemistry, Faculty of Science, University of Dschang, Dschang, Cameroon; Universiteit Antwerpen, BELGIUM

## Abstract

Although studies on African Trypanosomiases revealed a variety of trypanosome species in the blood of various animal taxa, animal reservoirs of *Trypanosoma brucei gambiense* and anatomical niches such as skin have been overlooked in most epidemiological settings. This study aims to update epidemiological data on trypanosome infections in animals from human African trypanosomiasis (HAT) foci of Cameroon. Blood and skin snips were collected from 291 domestic and wild animals. DNA was extracted from blood and skin snips and molecular approaches were used to identify different trypanosomes species. Immunohistochemical analyses were used to confirm trypanosome infections in skin snips. PCR revealed 137 animals (47.1%) with at least one trypanosome species in the blood and/or in the skin. Of these 137 animals, 90 (65.7%) and 32 (23.4%) had trypanosome infections respectively in the blood and skin. Fifteen (10.9%) animals had trypanosome infections in both blood and skin snip. Animals from the Campo HAT focus (55.0%) were significantly (X^2^ = 17.6; P< 0.0001) more infected than those (29.7%) from Bipindi. Trypanosomes of the subgenus *Trypanozoon* were present in 27.8% of animals while *T*. *vivax*, *T*. *congolense* forest type and savannah type were detected in 16.5%, 10.3% and 1.4% of animals respectively. *Trypanosoma b*. *gambiense* infections were detected in the blood of 7.6% (22/291) of animals. No *T*. *b*. *gambiense* infection was detected in skin. This study highlights the presence of several trypanosome species in the blood and skin of various wild and domestic animals. Skin appeared as an anatomical reservoir for trypanosomes in animals. Despite methodological limitations, pigs, sheep, goats and wild animals were confirmed as potential reservoirs of *T*. *b*. *gambiense*. These animal reservoirs must be considered for the designing of control strategies that will lead to sustainable elimination of HAT.

## Author summary

Human African Trypanosomiasis (HAT) is a neglected tropical disease affecting some sub-Saharan Africa countries. Although HAT elimination can be considered as achieved in some foci, the interruption of its transmission by 2030 remains a challenge. Animal reservoir and skin-dwelling trypanosomes have emerged as factors that can compromise sustainable elimination of HAT. It is in this light that *T*. *b*. *gambiense* infections and different trypanosome species were identified in blood and skin of domestic and wild animals from two HAT foci of the forest region of Cameroon. For this study, blood and skin snip samples were collected from domestic and wild animals. Molecular tools were used to identify different trypanosome species. Several trypanosome species including trypanosomes of the subgenus *Trypanozoon*, *T*. *vivax* and *T*. *congolense* were detected in the blood and skin of several animal taxa. Immuno-histochemical tools confirmed trypanosome infections in the skin. Pigs, sheep, goats and wild animals were confirmed as potential animal reservoirs of *T*. *b*. *gambiense*. Results of this study highlighted the necessity of considering animal reservoirs as well as skin-dwelling trypanosomes in the designing of new control strategies that will lead to the interruption of HAT transmission by 2030.

## Introduction

Human African trypanosomiasis (HAT) also known as sleeping sickness is a neglected tropical disease affecting people living in sub-Saharan Africa. Protozoan parasites of the genus *Trypanosoma* are the causative agent of HAT. These parasites are transmitted by tsetse flies. Two related forms of these parasites are responsible for human disease [1]. The chronic form of HAT is caused by *Trypanosoma brucei gambiense* (*T*. *b*. *gambiense*). This form occurs in Western and Central Africa and is responsible of about 97% of sleeping sickness cases from 2001–2020 [2]. The acute form of HAT occurs in Eastern and Southern Africa and is due to *T*. *b*. *rhodesiense*. It is responsible of 3% of HAT cases [2]. However, during the last two years, the number of *gambiense*-HAT cases has been drastically reduced while that of *rhodesiense-*HAT cases remained relatively stable, meaning that 85% of the currently reported HAT cases are now linked to *T*. *b*. *gambiense*. Control efforts implemented during the last decades reduced the incidence of *gambiense*-HAT down to 992 and 663 new cases in 2019 and 2020, respectively [2]. Although the first global target of the WHO goal was achieved with less than 2,000 cases of *gambiense*-HAT recorded in 2020, its sustainable elimination (zero transmission) by 2030 could be jeopardized by several factors as animal reservoirs [3].

Domestic and wild animals were found with *T*. *b*. *gambiense* infections in several HAT foci [4–12]. Although *T*. *b*. *gambiense* have been identified in animals, the role played by animals as reservoir remains poorly understood. In some HAT foci, *T*. *b*. *gambiense* was identified in both human and animals while in others, *T*. *b*. *gambiense* was not identified in animals [13]. In addition to this animal reservoir of *T*. *b*. *gambiense*, trypanosome infections have been recently highlighted in the skin of mammalian host [14]. Experimental studies have demonstrated that trypanosomes can be found in the dermis of mouse models [14,15]. Importantly, skin-dwelling trypanosomes have also been recently identified in HAT patients as well as in parasitological unconfirmed seropositive individuals from a HAT focus in Guinea [16]. During the last century, extravascular trypanosomes have been identified in several organs including eyes, brain, skeletal muscle, lungs, adipose tissue, heart and reproductive organs [17]. These tissue-resident parasites may be implicated in human and veterinary disease and are thought to contribute to several clinical aspects of African trypanosomiasis, such as heart disease, weight loss, and vision loss [17]. Although some cutaneous symptoms such as pruritus, rashes and skin lesions have been reported in mammals carrying trypanosome infections [18,19], the presence of skin-dwelling trypanosomes remains poorly documented in naturally infected mammals because skin infections have been overlooked. Filling this gap requires investigations targeting skin-dwelling trypanosomes in naturally infected mammals. Such investigations could provide additional data to better understand the epidemiological situation of both HAT and animal African trypanosomiasis (AAT).

AAT, also known as “Nagana”, is caused by trypanosome species including *T*. *b*. *brucei*, *T*. *congolense*, *T*. *vivax* and *T*. *simiae* [20]. Affecting more than 50 million cattle and 230 million small ruminants and inducing an annual economic loss estimated at about $1300 million [21], AAT remains a major constraint for livestock and agricultural development in many Sub-Saharan countries [22]. The sympatric circulation of animal and human pathogenic trypanosomes in different animal taxa of some west and central African HAT foci may hinder their correct identification, especially for trypanosomes of the subgenus *Trypanzoon* where no tool is able to unequivocally differentiate these species because they are morphological identical and have considerable genetic similarities [23, 24, 25]. This could therefore jeopardize sustainable elimination of HAT due to difficulties of identifying animal reservoir of human infective trypanosomes.

## Materials and methods

### Ethics statement

The protocol of this study was approved by the Ethics Committee of the Ministry of Public Health of Cameroon under the reference N° 2019/02/1143/CE/CNERSH/SP of February 21, 2019. The local administrative and traditional authorities as well as inhabitants of each HAT focus gave also their verbal approval after detailed explanation of the objectives of the study.

### Study area

This cross-sectional study was conducted in March 2018 and November 2019 in the Campo and Bipindi HAT foci of the south of Cameroon. The Campo (2°20’N, 9°52’E) HAT focus is located along the Atlantic Ocean and the Ntem River that separates Cameroon from Equatorial Guinea. The population density is low with less than one inhabitant per kilometer square [26]. In this focus, the vegetation is of forest type and the hydrographic network is dense with many rivers. Inhabitants of this locality practice agriculture, fishing, hunting and small-scale breeding of domestic animals such as pigs, goats and sheep. This area is rich in wildlife and is part of the “Campo Ma’an” National Park.

The Bipindi (3°2’N, 10°22’E) HAT focus is located at approximately 75 km of the Atlantic Ocean cost. It has an equatorial climate with an average temperature of 25.5°C. The population density is low with less than one inhabitant per kilometer square. Hunting, agriculture and small-scale farming of domestic animals such as goats, sheep, and pigs are the main activities practiced by the inhabitants of this locality. The Bipindi HAT focus is surrounded by hills and has a dense hydrographic network containing several rivers that cross cocoa plantations.

### Blood sampling and skin snips collection

Domestic animals such as pig, dog, goat and sheep were sampled in all villages. One day before each sampling, the objective of the study was explained to inhabitants and local authorities of each HAT focus. Thereafter, inhabitants were asked to restrain and/or keep their animals for the next day. All domestic animals that have spent at least one month in the village were sampled. From each animal, blood was collected from the jugular vein in goat and sheep, from saphenous vein in dog, and from cava vein in pig.

In addition to domestic animals, wild animals were also sampled because results of previous studies highlighted trypanosome infections and especially *T*. *b*. *gambiense* infections in some species [4–12]. For this wildlife, the sampling was limited to animal that were accidentally met during our field activities because we did not want to encourage hunting activities in these localities. In these wild animals, blood sample were collected by cardiac puncture by a member of the research team.

From each collected blood sample, 500 μL were spread in a single spot on Whatman grade 4 qualitative filter paper (110 mm diameter) that was dried and keep at 4°C in the field, and then at -20°C in the laboratory until DNA extraction.

Two skin snips were also collected from each animal. During the collection, a manual clipper was used to remove the hair. Then, two blood-free skin snips, having each a diameter of 2 mm and a depth of 2 mm were collected using the holtz forceps. Moreover, skin sections were performed on formalin-fixed paraffin-embedded (FFPE) biopsies because the reading of immunohistochemical slide was focused on the reticular dermis. One of these skin snips was stored at -20°C in 500 μL of RNAlater until the extraction of nucleic acids while the other was conserved in 1 mL of Paraformaldehyde 10% for immunohistochemical analyses.

### DNA extraction from skin snips and dry blood spot (DBS) on whatman paper

From whole blood spread on Whatman paper, DNA was extracted using “Nucleospin Triprep” kit following the manufacturer’s instructions. Briefly, from each filter paper containing spread blood, 5 pieces having 5 mm of diameter for each were introduced into microtube of 1.5 μL. Thereafter, 350 μL of buffer RP1 and 3.5 μL of β-mercaptoethanol were added to each microtube that was incubated at 65°C for 2 hours. During this incubation, microtubes were homogenized by vortexing every 30 min. The mixture was collected and applied on the violet ring NucleoSpin filter initially placed into a collection tube of 2 mL. The tubes were centrifuged at 11,000g for 1 minute. To the eluate, 350 μL of ethanol (70%) were added and the mixture was homogenized by pipetting up and down 5 times. The resulting mixture was introduced in the light blue ring NucleoSpin column placed in a new collection tube of 2 mL. After another centrifugation at 11,000g for 30s, each column was washed twice by adding 500 μL of DNA wash buffer followed by a centrifugation at 11,000 g for 1 minute. DNA was eluted by adding, to the column placed in 1.5 mL microtube, 80 μL of elution buffer. After this addition, the column was incubated at room temperature for 5 minutes before another centrifugation at 11,000 g for one minute. The resulting DNA extracts were stored at -20°C for molecular analyses.

Skin snips preserved in RNAlater were removed from the solution. Each skin snip was dried by incubation at 37°C for 30 minutes. Thereafter, DNA was extracted from each skin snip using the “Nucleospin Triprep” kit as describe above.

A negative extraction control was used during the DNA extraction to ensure that the contamination by trypanosome DNA does not occurs during the extraction process. For this negative extraction control, sterile distilled water was used and this water followed the whole extraction process. The qubit fluorometer was used to assess the amount of DNA in each DNA extract.

### Molecular identification of trypanosome species

This identification was done by PCR as described by Simo et al. [4] using specific primers for trypanosomes of the sub-genus *Trypanozoon*, *T*. *vivax*, *T*. *congolense* forest and savannah (Table 1). Each PCR reaction was carried out in a final volume of 15 μL containing 1.5 μL of 10X PCR reaction buffer, 1.5 mM MgCl_2_, 200 mM of each dNTP, 10 picomoles of each primer, 0.3 units of Taq DNA polymerase (New England Biolabs, 5 U/μL stock solution) and 3 μL of DNA extract. For each PCR reaction, a denaturation step at 94° C for 5 min was followed by 40 amplification cycles. Each of these cycles was made up of a denaturation step at 94° C for 30 s, an annealing step at 60° C for 30 s for *T*. *vivax*, *T*. *congolense* forest and savannah, and at 58° C for 1 min for trypanosomes of the sub-genus *Trypanozoon* and an extension step at 72° C for 1 min. A final extension step was performed at 72° C for 10 min.

**Table 1:** Sequence of the different primers and expected size.

Specificity	Code	Primers sequence	Expected size (bp)	References
*T*. *congolense* forêt	TCF1 TCF2	5’-GGACACGCCAGAAGTACTT-3’ 5’-GTTCTCGCACCAAATCCAAC-3’	350	[27]
*T*. *congolense* savannah	TCN1 TCN2	5’-TCGAGCGAGAACGGGCACTTTGCGA-3’ 5’-ATTAGGGACAAACAAATCCCGCACA-3’	341	[28]
*Vivax*	TVW1 TVW2	5’-CTGAGTGCTCCATGTGCCAC-3’ 5’-CCACCAGAACACCAACCTGA-3’	150	[27]
*Trypanozoon*	TBR1 TBR2	5’-GAATATTAAACAATGCGCAG-3’ 5’-CCATTTATTAGCTTTGTTGC-3’	164	[28]
*T*. *brucei gambiense*	TgsGp1 TgsGp2 TgsGps TgsGpas	5’GCT GCT GTG TTC GGA GAG C-3’ 5’-GCC ATC GTG CTT GCC GCT C-3’ 5’-TCA GAC AGG GCT GTA ATA GCA AGC-3’ 5’-GGG CTC CTG CCT CAA TTG CTG CA-3’	270	[29] [30]

After PCR reactions, 10 μL of amplified products were resolved by electrophoresis on 2% agarose gel that was subsequently stained with gel red, visualized under ultraviolet light and then photographed.

PCR reactions were performed twice for all positive samples. Each sample was considered positive for one trypanosome species only if this sample remained positive for the two PCRs. For *T*. *b*. *gambiense* infections, in addition to these two PCRs, its present was confirmed by the sequencing of TgsGP DNA fragments generated by the nested PCR.

### Molecular identification of *Trypanosoma brucei gambiense*

From TBR1/2 primers PCR data, all samples showing a DNA fragment at 164 bp corresponding to the expected size for trypanosomes of the sub-genus *Trypanozoon* were selected and subjected to the molecular identification of *T*. *b*. *gambiense*. This was performed using a nested PCR as described by Cordon-Obras et al. [6]. For this identification, two sets of primers were used [29, 30] (Table 1). The first PCR round was carried out in a total volume of 25 μL containing 1X PCR buffer [Tris– 10 mM HCl (pH 9.0), 50 mM KCl, 3 mM MgCl_2_], 15 picomoles of each primer (TgsGP-1/2), 100 mM of each dNTP, one unit of Taq DNA polymerase, 5 μL of DNA extract and 14 μL of sterile water. The amplification program contained an initial denaturation step at 95° C for 3 min followed by 45 cycles. Each of these cycles was made up of a denaturation step at 95° C for 30 s, an annealing step at 63° C for 1 min and an elongation step at 72° C for 1 min. A final elongation was done at 72° C for 5 min.

Amplicons of the first PCR were diluted 10 times and 5 μL of each dilution were subsequently used as DNA template for the second PCR in which TgsGPs/as primers were used. In this nested PCR, only 25 amplification cycles were performed in the same conditions as for the first PCR. For both reactions, two negative controls were used; a negative extraction control and a negative PCR control (from the first PCR).

Amplicons from the second PCR were resolved by electrophoresis on 2% agarose gel containing gel red (0.3μg/ml). The DNA bands were visualized and photographed under ultraviolet (UV) light. All samples with a 270-bp DNA fragment corresponding to the expected size for *T*. *b*. *gambiense* were selected for sequencing.

### Purification and sequencing of *T*. *b*. *gambiense* DNA fragments

All samples for which a DNA fragment of 270 bp was amplified were selected and then, newly amplified in a final volume of 50 μL. Amplicons were purified using the QIAquick PCR purification kit as described by the manufacturer. Purified amplicons were sequenced in both directions by a commercial company (Eurofins). The Basic Local Alignment Search Tool (BLAST) was used in NCBI to compare the sequences obtained with those available in the GenBank database. The alignments of these sequences were performed using ClustalW program. Considering the e-value and the percentage of identity, a sequence was assigned to *T*. *b*. *gambiense* when the e-value was equal to 2e-64 for both strands and the percentage of identity equal to 100%.

### Immunohistochemical detection of trypanosomes in the skin snip

Immunohistochemical detection of trypanosomes in skin biopsies was performed as described by Camara et al. [16]. Briefly, formalin-fixed paraffin-embedded skin biopsy sections were immunolabelled with either *T*. *brucei*–specific anti-ISG65 antibody (1/1000) or *T*. *brucei*–specific anti-CRD antibody (1/750) that target respectively the Invariant Surface Glycoprotein 65 expressed at the surface of the mammalian host stages of *T*. *brucei* s.l. [31] and the Cross-Reactive Determinant of Variable Surface Glycoproteins of the mammalian host stages of *T*. *brucei* s.l. [32]. Although anti-ISG65 antibody is specific to *T*. *brucei* as it targets the surface invariant glycoprotein 65 only present in trypanosomes of the subgenus *Trypanozoon*, the anti-CRD antibody recognizes the cross-reacting determinant region present at the junction between trypanosome surface proteins and their GPI anchor to the membrane. Hence, it can detect a number of surface proteins in *Leishmania* and *Trypanosoma* parasites, including *T*. *brucei*, *T*. *vivax* and *T*. *congolense*. On this basis, samples that were positive to both antibodies were considered as infected by at least *T*. *brucei* s.l. whereas those that were only positive to the anti-CRD antibodies were likely considered as having *T*. *vivax* and/or *T*. *congolense* infections, but not *T*. *brucei*.

All slides were counterstained with the hematoxylin, present in the secondary antibody polymer kit, which stains acidic structures like nucleic acid in blue. Entire slides were blindly and automatically pictured with a Zeiss Axioscan and assessed in the Zeiss Zen software. The presence/absence of parasites was blindly assessed and the positivity was defined by the presence of at least five clearly distinguishable parasites, defined by the presence of a strong specific labelling of a structure isolated in the dermal matrix with a size and a morphology compatible with a full length of trypanosome.

### Data analysis

Chi-squared test was used to compare the positivity rate of trypanosome infections between blood and skin snip, according to HAT foci and animal species. For animal species with sample size below 30, the McNemar test was used. The difference was considered significant when the p-value was lower than 0.05.

### Mapping of trypanosomes infections

During field surveys, the geographical coordinates of each sampling site were collected with a GPS. Using Qgis v.3.16 software, *T*. *b*. *gambiense* infections in both humans and animals were plotted and mapped according to village.

## Results

### Sampling distribution

For this study, 291 animals including 275 (94.5%) domestic animals and 16 (5.5%) wild animals were sampled: 200 (68.7%) in Campo and 91 in Bipindi (S1 Table). The 16 wild animals included four monkeys (*Cercopithecus cephus*), four antelopes (*Neotragus pygmaeus*), three blue duikers (*Cephalophus monticola*), two spotted genets (*Genetta servalina*), one Bruce’s daman (*Hyraxes*), one porcupine (*Atherurus africanus*) and one pangolin (*Manis tricuspis*). The 200 animals from Campo included 111 pigs, 39 goats, 21 dogs, 18 sheep and 11 wild animals collected in 10 villages. The 91 animals from the Bipindi HAT focus included 22 pigs, 37 goats, 27 sheep and 5 wild animals sampled in three villages.

### Trypanosome infection rate according to animal species

**Fig 1:**
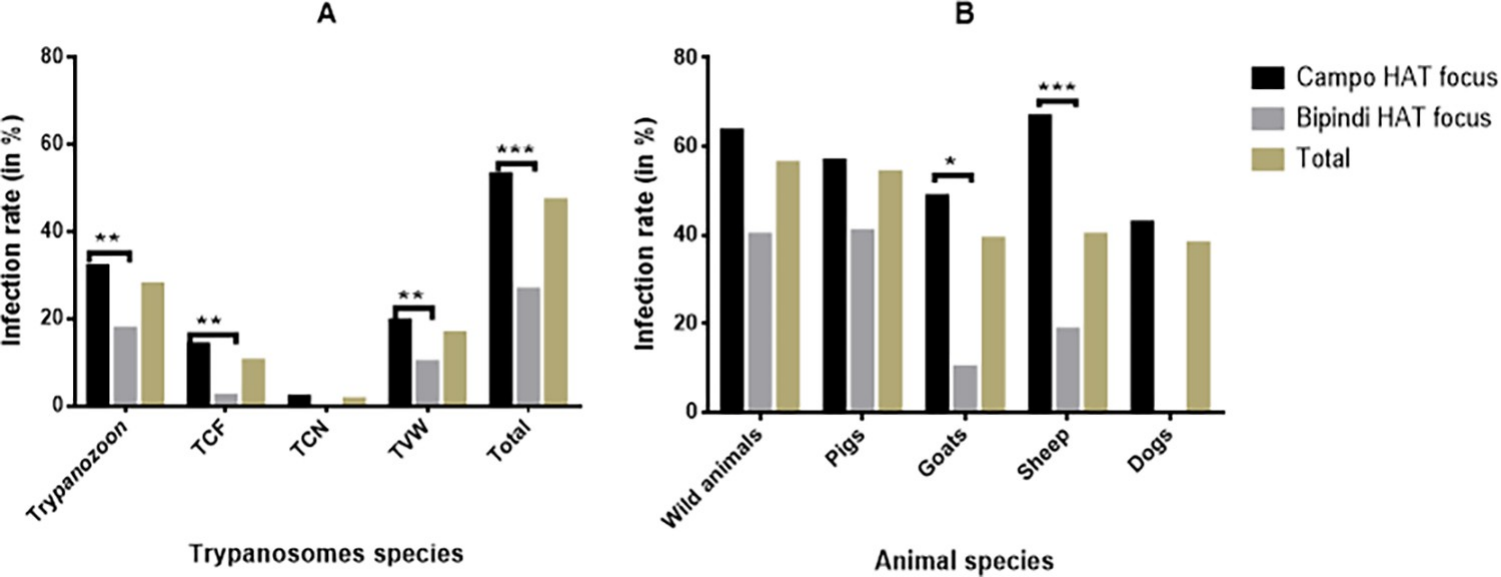
Overall trypanosome infection rates according to HAT focus, trypanosome species (A) and animal species (B). TCF: *T*. *congolense* forest; TCN: *T*. *congolense* savannah; TVW: *T*. *vivax*. *p-value = 0.05; **p-value<0.01; ***p-value<0.0001.

Among the 291 animals sampled, 137 (47.1%) carried at least one trypanosome species either in the blood or the skin (S1 Table). The highest trypanosome infection rate (56.2%; 9/16) was recorded in wild animals and the lowest infection rate (38.1%; 8/21) in dogs (Fig 1). Comparing the overall trypanosome infection rates, no significant difference (X^2^ = 6.5; p = 0.16) was observed between animal species. Trypanosomes of the subgenus *Trypanozoon*, *T*. *vivax*, *T*. *congolense* forest and savannah were recorded in all animal species. Significant differences (X^2^ = 89.6; p <0.0001) in infection rates were recorded between trypanosome species. Trypanosomes of the subgenus *Trypanozoon* were the most detected (27.8%; 81/291) in contrast to *T*. *congolense* savannah (1.4%; 4/291). More details about the distribution of trypanosome infections according to animal species and trypanosome species are shown in S2 Table. This pattern of trypanosome infections was observed in the two HAT foci (Fig 1).

### Trypanosome infection rates according to HAT foci

In the Campo HAT focus, the overall trypanosome infection rate was 55.0% (110/200). These infection rates ranged from 66.7% (12/18) for sheep to 38.1% (8/21) in dogs (Fig 1). Four species of trypanosomes including *T*. *brucei* s.l., *T*. *vivax*, *T*. *congolense* forest and *T*. *congolense* savannah were identified in this focus.

In Bipindi, the overall trypanosome infections rate was 29.7% (27/91). These infection rates ranged from 40.9% (9/22) for pigs to 22.2% (6/27) for sheep. Excepted for *T*. *congolense* savannah, *T*. *brucei* s.l., *T*. *vivax*, *T*. *congolense* forest were found in this focus (Fig 1).

Animals from the Campo HAT focus (55.0%) were significantly (X^2^ = 17.6; P < 0.0001) more infected than those from the Bipindi HAT focus (29.7%) (Fig 1).

### Trypanosome infection rates according to sample type (blood or skin) and HAT foci

Out of the 137 infected animals, 15 (10.9%) had trypanosome infections both in blood and skin while 90 (65.7%) and 32 (23.4%) had trypanosome only in blood or in skin, respectively. From the 291 animals analysed in the two HAT foci, trypanosome infections were recorded in the blood and skin of 105 (36.1%; 105/291) and 47 (16.2%; 47/291) animals respectively. Comparing the overall trypanosome infection rates in the blood and skin, significant differences were recorded in pigs (X^2^ = 17.3; p<0.0001), goats (X^2^ = 7.13; p = 0.008), sheep (X^2^ = 4.4; p = 0.04) and wild animals (X^2^ = 4.76; p = 0.029); trypanosome infections being significantly higher in the blood than the skin (Table 2). The infection rate of trypanosomes of the subgenus *Trypanozoon* was significantly (X^2^ = 36.8; p < 0.0001) higher in blood than the skin (Table 2). Except Mintom where only blood infections were identified in animals, blood and skin trypanosomes were identified in all villages. More details about the distribution of trypanosome infections between villages are given in S1 Table.

**Table 2:** Proportion of blood and skin-dwelling trypanosome infections according to animal species and HAT foci.

Animal species	Samples	Trypanosomes infections			
		Campo HAT focus		Bipindi HAT focus	
		NEA	TI (%)	NEA	TI (%)
Pig	Blood	111	49* (44.1)	22	6 (27.3)
	Skin	111	21* (18.9)	22	3* (13.6)
	Blood and/or skin	111	63*φ (56.7)	22	9 (40.9)
	X^2^		**16.3**		1.2
	P-value		**<0.0001**		0.26
Goat	Blood	39	16* (41.02)	37	9 (24.3)
	Skin	39	9* (23.1)	37	2 (5.4)
	Blood and/or skin	39	20*φ (51.3)	37	10*φ (27.02)
	X^2^		2.9		**5.2**
	P-value		0.09		**0.02**
Sheep	Blood	18	8* (44.4)	27	5 (18.5)
	Skin	18	4 (22.2)	27	1 (3.7)
	Blood and/or skin	18	12 (66.7)	27	6 (22.2)
	X^2^		2		3
	P-value		0.16		0.08
Dog	Blood	21	4* (19.05)	/	/
	Skin	21	5 (23.8)	/	/
	Blood and/or skin	21	8*φ (38.1)	/	/
	X^2^		0.14		
	P-value		0.71		
Wild animal	Blood	11	6* (54.5)	5	2 (40)
	Skin	10	2* (20)	5	0 (0)
	Blood and/or skin	11	7*φ (63.6)	5	2 (40)
	X^2^		2.6		2.5
	P-value		0.10		0.11
**Total**	Blood	200	83* (41.5)	91	22 (24.2)
	Skin	199	41* (20.6)	91	6* (6.6)
	**Blood and/or skin**	**200**	110*φ **(55)**	**91**	27*φ **(29.7)**
	**X** ^**2**^		**20.3**		**10.8**
	**P-value**		**<0.0001**		**0.001**

NEA: Number of Examined Animals; TI: Total of infected animals *: mixed infections or animal in which more than one trypanosome species was detected in the blood and/or the skin

φ: animal with trypanosome infections in both the blood and skin or animal in which trypanosomes were detected in both blood and skin

*φ: animal carrying more than one trypanosome species or having mixed infections and in which trypanosomes were detected in both blood and skin

X^2^: Chi square.

X^2^ and P-value refer to variations of blood and skin trypanosomes for each animal species and in each focus.

The proportion of infected animals was compared between blood and skin within each focus and animal species. Four species of trypanosomes including trypanosomes of the sub-genus *Trypanozoon*, *T*. *vivax*, *T*. *congolense* forest and *T*. *congolense* savannah were found in the skin of animal from Campo while only trypanosomes of the sub-genus *Trypanozoon* and *T*. *vivax* were recorded in those from Bipindi (S3 Table). The 47 animals from the Campo and Bipindi HAT foci that had skin trypanosomes included 5 (23.8%) dogs, 24 (18.04%) pigs, 11 (14.5%) goats, 5 (11.1%) sheep and two (20%) wild animals. Of these 47 animals with skin trypanosomes, significant differences (X^2^ = 19.7; p < 0.0001) were recorded in the infection rates of different trypanosome species; *T*. *vivax* (7.9%; 23/290) being the most prevalent species in the skin and *T*. *congolense* savannah (0.3%; 1/290) the less prevalent (S2 Table). Animals from the Campo HAT focus (20.6%; 41/199) harbored significantly (X^2^ = 9.02; p = 0.003) more skin trypanosome infections compared to those (6.6%; 6/91) from Bipindi (Table 2).

**Fig 2:**
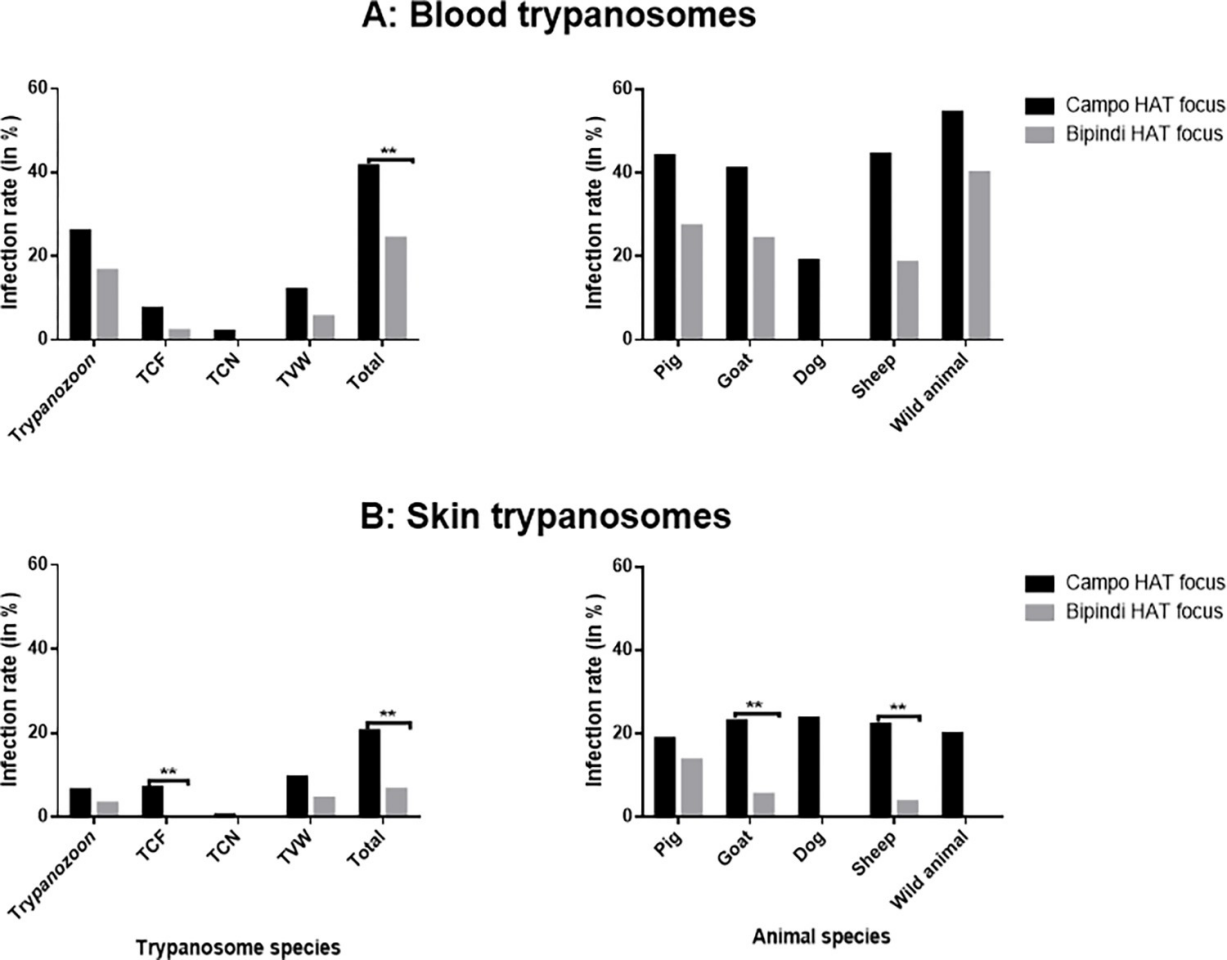
Trypanosomes infection rates in blood (A) or skin (B) according to HAT foci. TCF: *T*. *congolense* forest; TCN: *T*. *congolense* savannah; TVW: *T*. *vivax*. **p-value<0.05.

Out of the 41 animals found with skin trypanosomes in Campo, *T*. *vivax* had the highest prevalence (9.5%; 19/199) and *T*. *congolense* savannah (0.5%; 1/199) the lowest prevalence. The trypanosome infection rates varied significantly (X^2^ = 15.81; p = 0.001) between trypanosome species, but not between animal species (X^2^ = 0.5; p = 0.97). Dogs have the highest infection rate of 23.8% (5/21) and wild animals the lower infection rate of 20% (2/10) (Fig 2).

Six animals from Bipindi had skin trypanosomes: 3 pigs, 2 goats and one sheep. These animals were more infected by *T*. *vivax* (4.4%; 4/91) followed by trypanosomes of the subgenus *Trypanozoon* (3.3%; 3/91).

### Confirmation of cutaneous trypanosome infections by immunohistochemistry

**Fig 3:**
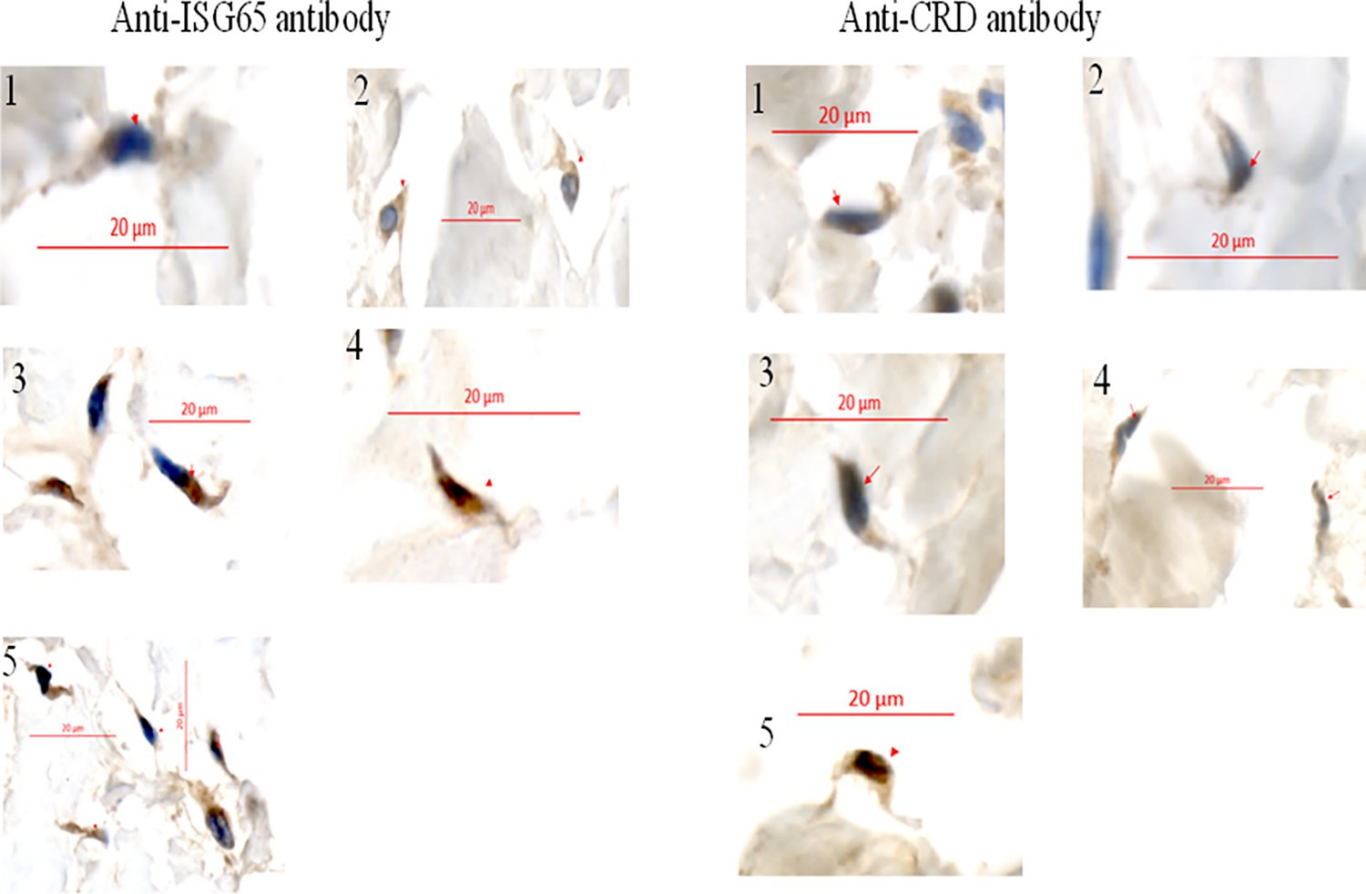
Extravascular trypanosomes in skin snip samples collected in animals. Each skin snip samples was stained with two antibodies: an anti-ISG65 antibody and an anti-CRD antibody. Numbers 1 to 5 represent the codes assigned to 5 samples stained with either anti-ISG65 antibody or anti-CRD antibody. Scale bars represent 20μm and red arrows indicate trypanosomes in the dermis.

To confirm the molecular data obtained from the skin, we selected 21 samples (15 pigs, 3 dogs, 2 sheep and 1 goat) for immunohistochemical analyses based on their positivity for trypanosomes of the sub-genus *Trypanozoon* and the presence of double and triple infections in the blood and/or the skin. From these 21 animals, skin trypanosomes were identified by PCR in 6 (28.6%) of them. Out of the 21 selected skin snip samples, 18 (85.7%; 18/21) were positive to at least one of the two antibodies (anti-ISG65 and/or anti-CRD antibodies). Among the 18 skin snips found with trypanosome infections, 10 (47.6%) were positive for the two antibodies and 8 (38.1%; 8/21) were positive to anti-CRD antibody only (Fig 3).

### Mixed infections of different trypanosomes species in either the blood or the skin

In the Campo HAT focus, 12 (6%) animals carried more than one trypanosome species in the blood. Five (2.5%) of them were co-infected with *T*. *congolense* forest and *T*. *vivax*, 3 (1.5%) with trypanosomes of the subgenus *Trypanozoon* and *T*. *congolense* savannah, 3 (1.5%) with trypanosomes of the subgenus *Trypanozoon* and *T*. *vivax* and one (0.5%) with *T*. *congolense* forest and trypanosomes of the subgenus *Trypanozoon*. Six (3%) animals had more than one trypanosome species in the skin: two (1%) had co-infections of trypanosomes of the subgenus *Trypanozoon* and *T*. *congolense* forest, 2 (1%) with trypanosomes of the subgenus *Trypanozoon* and *T*. *vivax* and the last 2 (1%) with *T*. *congolense* forest and *T*. *vivax*. In the Bipindi HAT focus, no mixed infection of different trypanosome species was identified in the blood. However, one co-infection involving trypanosomes of the subgenus *Trypanozoon* and *T*. *vivax* was detected in the skin of a pig.

### Trypanosome infection rates in both blood and skin

**Fig 4:**
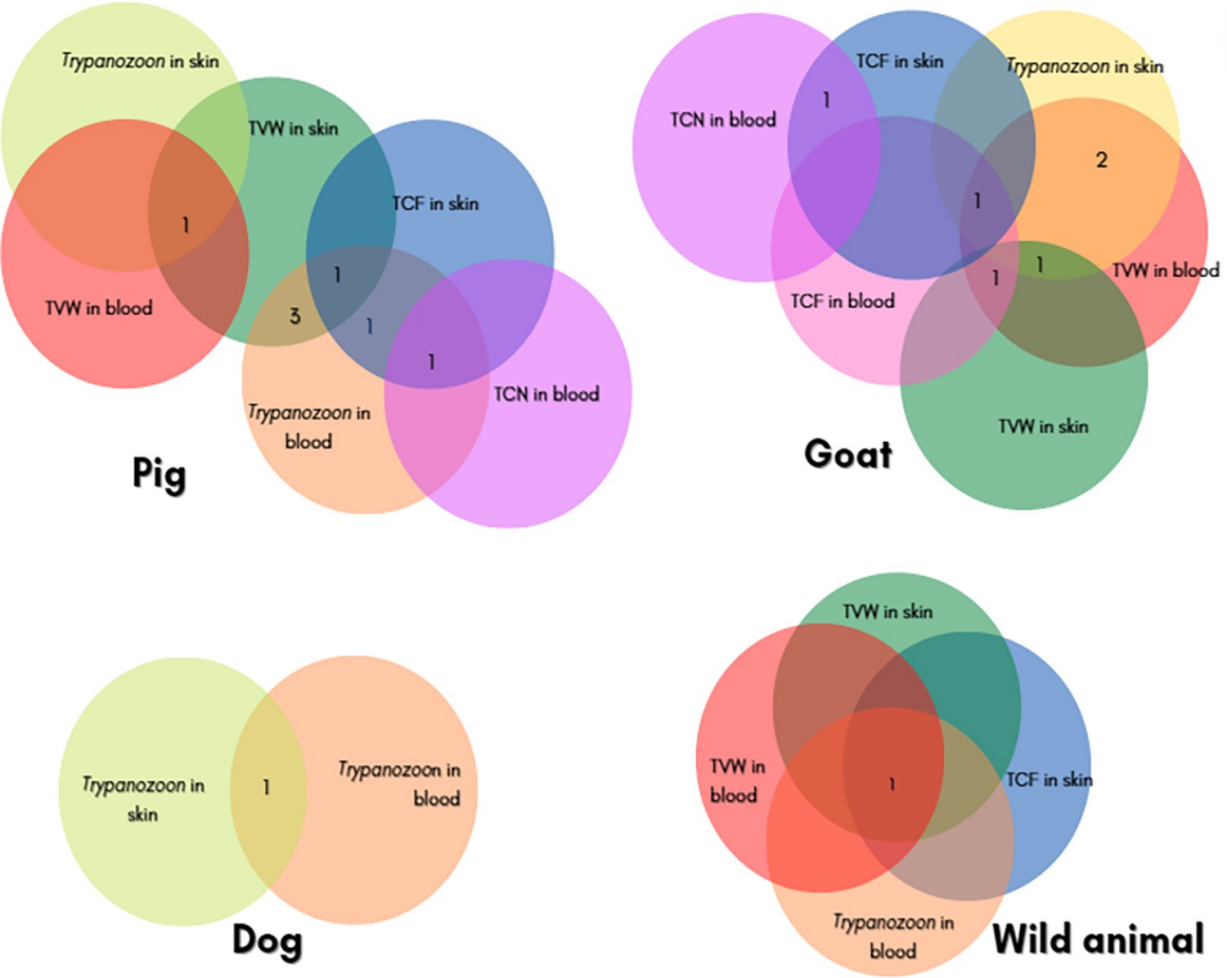
Mixed infections showing both blood and skin trypanosomes according to animal species. TCF: *T*. *congolense* forest type; TCN: *T*. *congolense* savannah type; TVW: *T*. *vivax*.

Fig 4 illustrates the complexity and the diversity of mixed infections found in blood and skin of each animal species. Fifteen (10.9%: 15/137) animals (7 pigs, 6 goats, one dog and one wild animal) harbored trypanosomes in both blood and skin. Three pigs had co-infections of *T*. *vivax* in the skin and trypanosomes of the subgenus *Trypanozoon* in blood while two other were co-infected by trypanosomes of the subgenus *Trypanozoon* in the blood and *T*. *congolense* either in the blood or skin. Two goats were co-infected by trypanosomes of the subgenus *Trypanozoon* in the skin and *T*. *vivax* in blood. One wild animal was co-infected by trypanosomes of the subgenus *Trypanozoon* in the blood, *T*. *congolense* in skin and *T*. *vivax* in both blood and skin. Of the 15 (10.9%: 15/137) animals carrying both blood and skin trypanosomes, 5 (3.6%: 5/137) had the same trypanosomes species both in the blood and skin while 10 (7.3%: 10/137) had different trypanosome species in the blood and in skin.

### *Trypanosoma brucei gambiense* infections in wild and domestic animals

Among the 66 animals with trypanosomes of the subgenus *Trypanozoon* in the blood, 22 (7.6%; 22/291) gave positive signal in the nested PCR for *T*. *b*. *gambiense* specific TgsGP: 15 animals from Campo and 7 from Bipindi. All the PCR products were sequenced and gave 100% identity with a TgsGP reference sequence (GenBank access number: FN555993). In the Campo HAT focus, *T*. *b*. *gambiense* infections were found in 8.1% (9/111) of pigs, 5.1% (2/39) of goats, 5.5% (1/18) of sheep and 27.3% (3/11) of wild animals (1 Pangolin and 2 Antelopes). No *T*. *b*. *gambiense* infection was recorded in dogs from this focus. The 15 animals from Campo that carried *T*. *b*. *gambiense* infections were recorded in 5 villages: 7 animals from Campo ville, 3 from Campo beach, two from Ipono, two from Tonde fan and one from Mintom.

The seven blood infections of *T*. *b*. *gambiense* of the Bipindi HAT focus included 5 (13.5%: 5/37) goats, one (3.7%: 1/27) sheep and one (20%: 1/5) wild animal (Spotted genet). *Trypanosoma b*. *gambiense* infections were found in animals from all villages of the Bipindi HAT focus: 4 infections in animals from Bidjouka, 2 in those from Lambi and one in animal from Bipindi centre.

No *T*. *b*. *gambiense* infection was detected in the skin of animals.

### Mapping animal *T*. *b*. *gambiense* infections with HAT cases

**Fig 5:**
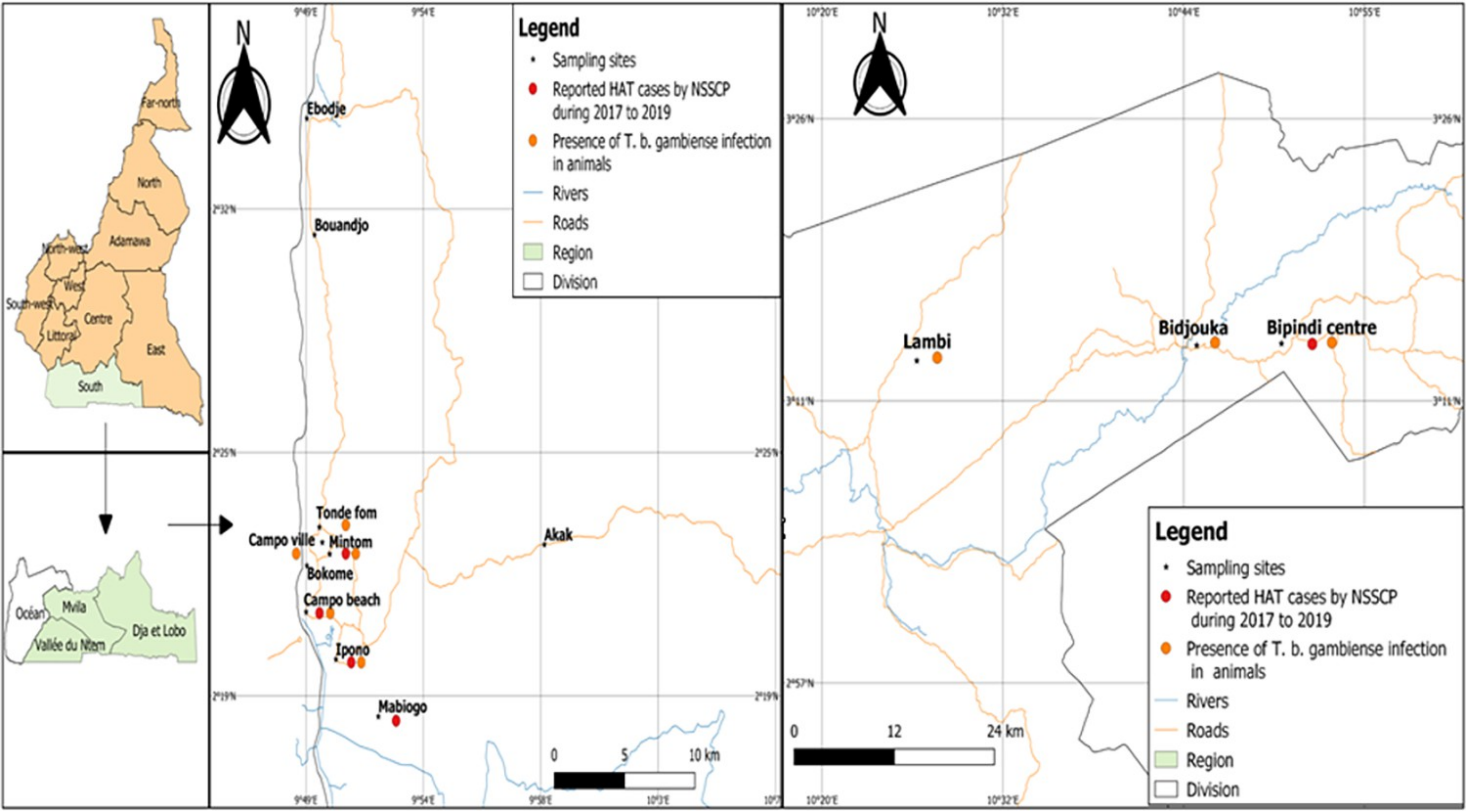
Spatial distribution of *T*. *b*. *gambiense* infection in animals and HAT cases in Campo (left) and Bipindi (right) HAT foci. https://gadm.org/download country v3.html.

Data on human infections were collected from the National sleeping Sickness Control Program (NSSCP) of Cameroon. These data revealed that 28 HAT cases were recorded in 5 villages from 2017 to 2020. Fig 5 shows spatial distribution of these cases as well as *T*. *b*. *gambiense* infections. From these 5 villages, *T*. *b*. *gambiense* infections were recorded in animals from 4 of them: Ipono, Mintom, Campo beach and Bidjouka. In Mabiogo where 8 HAT cases were diagnosed between 2017 and 2020, no *T*. *b*. *gambiense* infection was recorded in animals.

## Discussion

Although most studies on African trypanosomes highlighted the circulation of trypanosomes in the blood of mammalian hosts, no study attempted to detect skin trypanosomes and assessed their infection rates in naturally infected domestic and wild animals. To fill this gap, this study was conducted in the Campo and Bipindi HAT foci of Cameroon in order to update epidemiological data on AAT and animal reservoir of *T*. *b*. *gambiense* by determining the infection rates of blood and skin trypanosomes in various animal taxa.

The identification of different trypanosome species in the blood of various animal taxa from the HAT foci of Campo and Bipindi is in agreement with results of previous authors reporting trypanosomes of the subgenus *Trypanozoon*, *T*. *congolense* and *T*. *vivax* in domestic and wild animals of these HAT foci [4,23,24,33–36]. These results confirm the circulation of different trypanosome species in wild and domestic animals of HAT foci of the forest regions of Cameroon. These observations could be mitigated because no parasitological and/or immunological method was used to confirm the presence of trypanosome infections. Indeed, a positive PCR indicates the presence of trypanosome DNA and does not necessarily reflect an active infection. Moreover, the problems of the reproducibility of PCR results in the detection of trypanosome infections have been already highlighted [37].

The trypanosome species identified in animals from the Campo HAT focus are in line with results recently obtained in tsetse flies captured the same year in the same HAT focus [25]. The overall trypanosome infection rate of 47.1% obtained in the present study is higher than the 31.3% and 23% previously reported in the Campo and Bipindi HAT foci [8]. This higher infection rate could be explained by the fact that both blood and skin trypanosomes were considered while in the previous studies, only blood trypanosome infections were detected. Nevertheless, it is important to mention that our infection rate of 36.1% recorded in blood is closed to the 31.3% reported about ten years ago in the same HAT foci [8]. These high trypanosome infection rates in animals from the two HAT foci could be explained by the fact that no effective control measures against AAT have been implemented in these HAT foci before our sampling. Moreover, as these HAT foci are located in the forest regions, the movements of domestic and wild animals between villages and the forest create conditions in which tsetse flies can acquire trypanosome infections from wild animals and then, transmit them to domestic animals. This hypothesis is strengthened by the high infection rates of the same trypanosome species that have been continuously recorded in tsetse flies as well as in wild and domestic animals of these HAT foci [5,38].

Although the anaemic status of sampled animals was not determined, most of these animals were apparently healthy because the veterinarian who clinically assessed all animals during field surveys reported no clinical signs related to AAT such as fever, weakness, lethargy or deterioration of physical conditions. This could be explained by the fact that most of sampled animals were pigs and small ruminants that do not stay for long in these HAT foci. Having a short life expectancy of less than 2 years due to the fact that most of these animals are generally sale for family purposes (funerals, scolarity, medical care), the clinical signs characterizing trypanosome infections or AAT cannot appear in most animals. In addition to that, it is acknowledged that pigs and small ruminants including goats and sheep are trypanotolerant to several trypanosomes species including T. congolense and T. vivax and to some extend T. brucei s.l. [39].

The higher trypanosome infection rate recorded in animals of Campo compared to those of Bipindi could be explained by the density of tsetse flies that is higher in Campo than Bipindi [25,38]. Our results showing a predominance of trypanosomes of the subgenus *Trypanozoon* are in agreement with those of Herder et al. [33]. Although the infection rate of 56.2% obtained in the present study for wild fauna is higher than the value reported by Njiokou et al. [5], our data must be taken with caution due to our small sample size. The high trypanosome infection rate (54.1%) recorded in pigs could be explained by previous observations reporting the tsetse flies feeding preference for pigs compared to other animal taxa [38,40]. Under such circumstance, the probability of pigs to acquire trypanosome infections is higher compared to other animal taxa.

This study highlighted several trypanosome species in the skin of naturally infected domestic and wild animals using both molecular and immunohistochemical methods. These results are in agreement with those from animal models where trypanosome infections were detected in the skin of experimentally infected animals [14,15]. They highlight the possibility for mammalian skin to serve as reservoir for trypanosomes. This hypothesis is supported by previous observations indicating that trypanosome infections begin in mammalian hosts when tsetse mouthparts enter the skin to inject infected saliva during a blood meal [15]. During this process, trypanosomes contained in this saliva are deposited by tsetse flies in the dermal layer where they initiate the infection and can remain over its course [14,15]. These trypanosomes may play a role in the transmission of different trypanosome species [41]. Indeed, during experimental studies, Capewell et al. [14] demonstrated that not only substantial quantities of trypanosomes exist within the skin, but also that these parasites can be transmitted to tsetse flies even in the absence of detectable parasitaemia. This indicates that the dermal trypanosomes identified in animals of our study can be transmitted to tsetse flies during their blood meals. Knowing that having accurate disease prevalence is one of the key components for the planing of effective control of African trypanosomiasis, our results show that in addition to the identification of trypanosome infections in the blood, there is a need to also determine the prevalence of skin trypanosomes. Combining data on trypanosome infections in the blood and skin will enable to have the real prevalence of these parasites in mammals. Such data are of great importance for the designing of efficient control measures and also for the monitoring and evaluation of control programs. The identification of trypanosomes of the sub-genus *Trypanozoon* in animal skin is not surprising because *T*. *b*. *brucei* and its related subspecies are tissular parasites that have been reported in the skin [42,43]. However, previous studies described *T*. *congolense* as completely intravascular parasite [44,45] and its identification in the skin contrasts previous observations [18,46]. Our results identifying *T*. *congolense* in the skin could be explained by the use of molecular tools which are more sensitive compared to microscopic observations used in previous studies. The presence of *T*. *vivax* and *T*. *congolense* in animal skin is in agreement with previous observations from experimental studies [18,46]. Indeed, an association has been reported between the presence of parasites living in the skin and local skin reactions elicited in animals bitten by tsetse flies infected with *T*. *congolense* or *T*. *vivax* [18,46]. Moreover, experimental studies reported a pattern of skin reactions at the site of infection and associated it with the presence of trypanosome species such as *T*. *congolense* or *T*. *vivax* [18,46].

Results of the present study show that some animals had trypanosome infections in the skin, but not in the blood while most animals had only blood infections. The high infection rate of blood trypanosomes compared to dermal ones indicates that the probability of having blood trypanosomes contaminating skin was unlikely in our experiment design. Although our cross-sectional study does not enable to have an idea about the becoming of trypanosomes detected in the skin and/or the blood of different animal taxa, speculations can be raised about: i) the tissues tropism of some trypanosome species; ii) the sensitivity of diagnostic tools (are these tools efficient enough for dermal trypanosomes?) and; iii) the biological dynamics of trypanosomes between blood and skin. Better understanding these factors requires additional investigations aiming not only to genetically characterize trypanosomes circulating in the blood and/or skin, but also to follow up the becoming of skin or blood trypanosome infections. This study showed that about 23.4% of trypanosome infections recorded only in animals’ skin will remain undetected using conventional blood-based approaches. This suggests that dermal trypanosomes have been largely overlooked during investigations on African trypanosomiases. Parasites hidden in the skin are potential reservoirs that could sustain the transmission of different trypanosome species. Moreover, their sensitivity to trypanocides is still unknown. In this context, the real disease prevalence remains unknown. Understanding the actual situation would be important for the designing of appropriate control measures against African trypanosomiasis.

Immunohistochemical analyses confirmed the presence of dermal trypanosomes in domestic animals of Campo. The fact that some skin snips were negative by molecular detection, but positive by immunohistochemistry highlights the need to develop more sensitive diagnostic tools to better identify trypanosome infections in the skin. Moreover, the need to have or develop specific antibodies for *T*. *vivax* and *T*. *congolense* is also of great interest to understand not only the lack of congruence in the detection of blood and skin trypanosomes in the same mammal, but also in the context of co-infections where inter-species interactions and associations with skin trypanosomes need to be well described. Developing such tools is essential not only for accurate determination of the disease prevalence, but also for a better understanding of the real epidemiological situation of trypanosome infections in different bio-ecological settings. Such tools will also enable the monitoring and evaluation of control programs that will lead to sustainable elimination of African trypanosomiasis.

The identification of *T*. *b*. *gambiense* in domestic and wild animals not only confirms results of previous authors [5,8,47], but also highlights a continuous circulation of *T*. *b*. *gambiense* in animals of these HAT foci. These animals could be considered as reservoirs of *T*. *b*. *gambiense* and they could maintain these parasites and ensure their low circulation in mammals. The identification of *T*. *b*. *gambiense* mainly in pigs is in agreement with previous data [4,8]. This result could be explained by the fact that tsetse flies feed preferentially on pigs rather than other animal taxa [38,40]. The identification of *T*. *b*. *gambiense* in animals of Campo is in line with result of Melachio et al. [25] who reported, between 2018 and 2019, the presence of human-infective trypanosomes in tsetse flies caught in this HAT focus. These results indicate active transmission of *T*. *b*. *gambiense* between humans, various animal taxa and tsetse flies. The infection rate of 7.6% recorded for *T*. *b*. *gambiense* in the Campo and Bipindi foci where few human cases were diagnosed could be explained not only by the lack of specificity of the nested PCR TgsGp used in this study, but also by the low level of anthropophilia of tsetse flies in these HAT foci. This low anthropophilia has been already highlighted by results of Melachio et al. [25]. Between 2018–2019 in the Campo HAT focus, these authors identified only two human bloodmeals out of 85 bloodmeals that were collected in *G*. *palpalis palpalis*. These results indicate that tsetse flies of these HAT foci feed most often on other vertebrate hosts like wild animals, pigs, goats, sheep [38]. In such context, there is the possibility of having transmission cycles including mainly domestic and wild animals as already reported in these HAT foci by previous studies [38]. Our results showing no infection of *T*. *b*. *gambiense* in animal skin could be explained by: i) the low sensitivity of the PCR target; ii) the low parasite load of *T*. *b*. *gambiense* in the skin and; iii) the small size of the biopsy samples.

The mapping of *T*. *b*. *gambiense* infections in animals and humans showed that both infections were recorded in some villages like Campo beach, Ipono, Mintom and Bipindi centre. These villages can be considered as those reporting the transmission cycle involving humans, animals and tsetse flies as suggested by Simo et al. [38]. They can also be considered as *T*. *b*. *gambiense* transmission hotspots where intensive vector control should be deployed. These results suggest that reaching the interruption of HAT transmission in 2030 as indicated in the Road map of the WHO would require to carefully consider animal reservoirs of *T*. *b*. *gambiense*. However, in villages like Tonde fam, Campo ville in the Campo HAT focus as well as Bidjouka and Lambi in the Bipindi HAT focus, *T*. *b*. *gambiense* infections were found in animals and no HAT cases was reported. These villages can be considered as those in which the transmission cycle may involve tsetse flies and animals. This type of transmission cycle has already been reported in some Central African HAT foci [6,7,11,38,48]. In Mabiogo where HAT cases were reported without *T*. *b*. *gambiense* infections in animals, the transmission cycle may involve tsetse flies and humans only. Nonetheless, the probability for inhabitants of Mabiogo to be infected in the forest cannot be ruled out due to their frequent movements between the villages and the forest. The results obtained in Mabiogo could also be justified by the small sample size.

Although the presence of *T*. *b*. *gambiense* in animals is in agreement with results obtained in tsetse flies caught during the same year in the same locality [25], its high prevalence is surprising. As already mentioned above, this high prevalence could result from the fact that a positive PCR does not necessarily reflect an active infection. In addition, as the genomes of *T*. *b*. *brucei* and *T*. *b*. *gambiense* are closely related, the risk for a nested-PCR to detect DNA fragments of *T*. *b*. *brucei* instead of *T*. *b*. *gambiense* cannot be excluded. Results of this study highlight again the need of developing more sensitive and specific markers for specific identification of *T*. *b*. *gambiense* infections in animals and tsetse flies [12,49].

## Conclusion

This study highlights the presence of several trypanosome species in the blood and skin of different wild and domestic mammal species in the Bipindi and Campo HAT foci. Animal skin can be considered as an anatomical reservoir for trypanosomes. AAT remains a serious threat for animal breeding in these forest areas. The natural infections of *T*. *b*. *gambiense* recorded in pigs, sheep, goats and wild animals like pangolins and antelopes confirmed their potential role of reservoir hosts for *T*. *b*. *gambiense* in these HAT foci of Cameroon. This study points out the need to consider hidden reservoirs of trypanosomes (skin-dwelling trypanosomes and animal reservoirs of *T*. *b*. *gambiense)* for the designing of optimal control strategies that will lead to sustainable elimination of African trypanosomiases.

## Supporting information

S1 Table Trypanosome infection rates by animal species, HAT foci and villages. NEA: Number of examined animals; NIA: number of infected animals; * mixed infections; ** both blood and skin trypanosome
(DOCX)

S2 Table Proportion of blood and skin-dwelling trypanosome infections according to animal species and trypanosomes species. NEA: Number of Examined Animals; *Trypanozoon*: *Trypanosoma brucei s*.*l;* TCF: *Trypanosoma Congolense* forest type; TCN: *Trypanosoma congolense* savannah type; TVW: *Trypanosoma vivax*, * mixed infections; ** both blood and skin trypanosomes. The proportion of infected animals was compared between animal species, trypanosomes species and between blood and skin sample.
(DOCX)

S3 Table Proportion of blood and skin-dwelling trypanosomes according to animal species, HAT foci and trypanosomes species. NEA: Number of Examined Animals; *Trypanozoon*: *Trypanosoma brucei s*.*l;* TCF: *Trypanosoma Congolense* forest type; TCN: *Trypanosoma congolense* savannah type; TVW: *Trypanosoma vivax*, * mixed infections; ** both blood and skin trypanosomes.
(DOCX)
