# Supplementary material for: Prevalence of blood and skin trypanosomes in domestic and wild fauna from two sleeping sickness foci in Southern Cameroon
Source: PLoS Negl Trop Dis. 2023 Jul 27;17(7):e0011528. doi: 10.1371/journal.pntd.0011528 (PMC10411957; doi:10.1371/journal.pntd.0011528)
Supplement: S1 Table — NEA: Number of examined animals; NIA: number of infected animals; * mixed infections; ** both blood and skin trypanosome (DOCX) [file pntd.0011528.s001.docx]

**Table S1 : Trypanosome infection rates by animal species, HAT foci and villages**

| **HAT foci** | **Villages** | **Animal species and infections rate** | | | | | | | | | | **Total** | |
| --- | --- | --- | --- | --- | --- | --- | --- | --- | --- | --- | --- | --- | --- |
|  |  | **Pig** | | **Goat** | | **Dog** | | **Sheep** | | **Wild animal** | |  |  |
|  |  | **NEA** | **NIA (%)** | **NEA** | **NIA (%)** | **NEA** | **NIA (%)** | **NEA** | **NIA (%)** | **NEA** | **NIA (%)** | **NEA** | **NIA (%)** |
| Campo | Campo ville | 44 | 22** (50) | 2 | 2 (100) | 0 | 0 (0) | 1 | 1 (100) | 8 | 5** (62.5) | 55 | 30** (54.5) |
|  | Ipono | 41 | 24** (58.5) | 5 | 5** (100) | 0 | 0 (0) | 0 | 0 (0) | 0 | 0 (0) | 46 | 29** (63.04) |
|  | Campo beach | 24 | 15* (62.5) | 1 | 1** (100) | 2 | 2 (100) | 0 | 0 (0) | 0 | 0 (0) | 27 | 18** (66.7) |
|  | Tonde fan | 0 | 0 (0) | 19 | 4* (21.1) | 0 | 0 (0) | 7 | 5 (71.4) | 0 | 0 (0) | 26 | 9** (34.6) |
|  | Mabiogo | 2 | 2 (100) | 9 | 7** (77.8) | 0 | 0 (0) | 0 | 0 (0) | 2 | 1 (50) | 13 | 10** (76.9) |
|  | Mintom | 0 | 0 (0) | 2 | 1 (50) | 2 | 1 (50) | 9 | 5 (55.6) | 0 | 0 (0) | 13 | 7 (53.8) |
|  | Bouandjo | 0 | 0 (0) | 0 | 0 (0) | 9 | 2** (22.2) | 1 | 1 (100) | 1 | 1* (100) | 11 | 4** (36.4) |
|  | Akak | 0 | 0 (0) | 0 | 0 (0) | 7 | 3* (42.8) | 0 | 0 (0) | 0 | 0 (0) | 7 | 3* (42.9) |
|  | Ebodje | 0 | 0 (0) | 0 | 0 (0) | 1 | 0 (0) | 0 | 0 (0) | 0 | 0 (0) | 1 | 0 (0) |
|  | Bokombe | 0 | 0 (0) | 1 | 0 (0) | 0 | 0 (0) | 0 | 0 (0) | 0 | 0 (0) | 1 | 0 (0) |
|  | **Total** | **111** | **63 (56.7)** | **39** | **20 (51.3)** | **21** | **8 (38.1)** | **18** | **12 (66.7)** | **11** | **7 (63.6)** | **200** | **110 (55)** |
| Bipindi | Bidjouka | 7 | 2 (28.6) | 15 | 4 (26.7) | 0 | 0 (0) | 16 | 3 (18.8) | 5 | 2 (40) | 43 | 11 (25.6) |
|  | Lambi | 4 | 1 (25) | 5 | 3* (60) | 0 | 0 (0) | 8 | 2 (25) | 0 | 0 (0) | 24 | 6* (25) |
|  | Bipindi centre | 11 | 6* (54.5) | 17 | 3 (17.6) | 0 | 0 (0) | 3 | 1 (33.3) | 0 | 0 (0) | 24 | 10* (41.7) |
|  | **Total** | **22** | **9* (40.9)** | **37** | **10* (27.02)** | **0** | **0 (0)** | **27** | **6 (22.2)** | **5** | **2 (40)** | **91** | **27 (29.7)** |
| **Total** |  | **133** | **72 (54.1)** | **76** | **30 (39.5)** | **21** | **8 (38.1)** | **45** | **18 (40)** | **16** | **9 (56.3)** | **291** | **137 (47.1)** |

NEA: Number of examined animals; NIA: number of infected animals; * mixed infections; ** both blood and skin trypanosome
