# Supplementary material for: Prevalence of blood and skin trypanosomes in domestic and wild fauna from two sleeping sickness foci in Southern Cameroon
Source: PLoS Negl Trop Dis. 2023 Jul 27;17(7):e0011528. doi: 10.1371/journal.pntd.0011528 (PMC10411957; doi:10.1371/journal.pntd.0011528)
Supplement: S2 Table — NEA: Number of Examined Animals; Trypanozoon: Trypanosoma brucei s.l; TCF: Trypanosoma Congolense forest type; TCN: Trypanosoma congolense savannah type; TVW: Trypanosoma vivax, * mixed infections; ** both blood and skin trypanosomes. The proportion of infected animals was compared between animal species, trypanosomes species and between blood and skin sample. (DOCX) [file pntd.0011528.s002.docx]

**Table S2:** Proportion of blood and skin-dwelling trypanosome infections according to animal species and trypanosomes species

| **Animal species** | **Samples** | **NEA** | **Number of infected animals (%)** | | | | **Total in blood or skin** | **Total in both blood and skin** | **X^2^** | **P-value** |
| --- | --- | --- | --- | --- | --- | --- | --- | --- | --- | --- |
|  |  |  | ***Trypanozoon*** | **TCF** | **TCN** | **TVW** |  |  |  |  |
| Pig | Blood | 133 | 36 (27.1) | 9 (6.8) | 1 (0.7) | 14 (10.5) | 55* (41.3) | **72** (54.1)** |  |  |
|  | Skin | 133 | 3 (2.2) | 8 (6.01) | 1 (0.7) | 15 (11.3) | 24* (18.04) |  |  |  |
|  | Total | 133 | 39 (29.3) | 17 (12.8) | 1** (0.7) | 28** (21.05) |  |  | **44.2** | **<0.0001** |
|  | X^2^ |  | **32.7** | 0.06 | 0 | 0.04 | **17.30** |  |  |  |
|  | P-value |  | **<0.0001** | 0.18 | 1.00 | 0.84 | **<0.0001** |  |  |  |
| Goat | Blood | 76 | 13 (17.1) | 3 (3.9) | 1 (1.3) | 10 (13.1) | 25* (32.9) | **30** (39.5)** |  |  |
|  | Skin | 76 | 8 (10.5) | 2 (2.6) | 0 (0) | 4 (5.3) | 11* (14.5) |  |  |  |
|  | Total | 76 | 20** (26.3) | 4** (5.3) | 1 (1.3) | 12** (15.8) |  |  | **26.9** | **<0.0001** |
|  | X^2^ |  | 1.38 | 0.21 | 1 | 2.83 | **7.13** |  |  |  |
|  | P-value |  | 0.24 | 0.65 | 0.32 | 0.09 | **0.008** |  |  |  |
| Sheep | Blood | 45 | 10 (22.2) | 1 (2.2) | 1 (2.2) | 2 (4.4) | 13* (28.9) | **18* (40)** |  |  |
|  | Skin | 45 | 1 (2.2) | 2 (4.4) | 0 (0) | 2 (4.4) | 5 (11.1) |  |  |  |
|  | Total | 45 | 11 (24.4) | 3 (6.7) | 1 (2.2) | 4 (8.9) |  |  | **13.3** | **0.004** |
|  | X^2^ |  | **8.4** | 0.34 | 1.01 | 0 | **4.4** |  |  |  |
|  | P-value |  | **0.004** | 0.56 | 0.31 | 1.00 | **0.04** |  |  |  |
| Dog | Blood | 21 | 3 (14.3) | 1 (4.8) | 1 (4.8) | 0 (0) | 4* (19.05) | **8** (38.1)** |  |  |
|  | Skin | 21 | 4 (19.05) | 1 (4.8) | 0 (0) | 0 (0) | 5 (23.8) |  |  |  |
|  | Total | 21 | 6** (28.6) | 2 (9.5) | 1 (4.8) | 0 (0) |  |  | **10.3** | **0.016** |
|  | X^2^ |  | 0.17 | 0 | 1.02 | / | 0.14 |  |  |  |
|  | P-value |  | 0.68 | 1.00 | 0.31 | / | 0.71 |  |  |  |
| Wild animal | Blood | 16 | 5 (31.2) | 3 (18.7) | 0 (0) | 3 (18.7) | 8* (50) | **9** (56.2)** |  |  |
|  | Skin | 15 | 0 (0) | 1 (6.7) | 0 (0) | 2 (13.3) | 2* (13.3) |  |  |  |
|  | Total | 16 | 5 (31.2) | 4 (25) | 0 (0) | 4** (25) |  |  | 5.7 | 0.13 |
|  | X^2^ |  | 5.59 | 1.01 | / | 0.17 | **4.76** |  |  |  |
|  | P-value |  | **0.018** | 0.32 | / | 0.68 | **0.029** |  |  |  |
| **Total** | Blood | 291 | 67 (23.02) | 17 (5.8) | 4 (1.4) | 29 (9.96) | 105* (36.1) | **137** (47.1)** | **84.1** | **<0.0001** |
|  | Skin | 290 | 16 (5.5) | 14 (4.8) | 1 (0.34) | 23 (7.9) | 47* (16.2) |  | **19.65** | **0.000** |
|  | **Total** | **291** | **81**(27.8)** | **30**(10.3)** | **4** (1.4)** | **48** (16.5)** |  |  | **89.6** | **<0.0001** |
|  | **X^2^** |  | **36.8** | 0.29 | 1.80 | 0.74 | **30.2** |  |  |  |
|  | **P-value** |  | **<0.0001** | 0.59 | 0.18 | 0.39 | **<0.0001** |  |  |  |

NEA: Number of examined animals; * mixed infections; ** both blood and skin trypanosome
