# Supplementary material for: Prevalence of blood and skin trypanosomes in domestic and wild fauna from two sleeping sickness foci in Southern Cameroon
Source: PLoS Negl Trop Dis. 2023 Jul 27;17(7):e0011528. doi: 10.1371/journal.pntd.0011528 (PMC10411957; doi:10.1371/journal.pntd.0011528)
Supplement: S3 Table — NEA: Number of Examined Animals; Trypanozoon: Trypanosoma brucei s.l; TCF: Trypanosoma Congolense forest type; TCN: Trypanosoma congolense savannah type; TVW: Trypanosoma vivax, * mixed infections; ** both blood and skin trypanosomes. (DOCX) [file pntd.0011528.s003.docx]

**Table S3: Proportion of blood and skin-dwelling trypanosomes according to animal species, HAT foci and trypanosomes species**

| **Animal species** | **HAT foci** | **Samples** | **NEA** | **PCR-positive result (Iinfection rate in %)** | | | | **Total in blood or skin** | **Total in both blood and skin** |
| --- | --- | --- | --- | --- | --- | --- | --- | --- | --- |
|  |  |  |  | ***Trypanozoon*** | **TCF** | **TCN** | **TVW** |  |  |
| Pigs | Campo | Blood | 111 | 33 (29.7) | 8 (7.2) | 1 (0.9) | 12 (10.8) | 49* (44.1) | **72* (54.1)** |
|  |  | Skin | 111 | 2 (1.8) | 8 (7.2) | 1 (0.9) | 12 (10.8) | 21* (18.9) |  |
|  | Bipindi | Blood | 22 | 3 (13.6) | 1 (4.5) | 0 (0) | 2 (9.1) | 6 (27.3) |  |
|  |  | Skin | 22 | 1 (4.5) | 0 (0) | 0 (0) | 3 (13.6) | 3* (13.6) |  |
|  | Total | Blood | 133 | 36 (27.1) | 9 (6.8) | 1 (0.7) | 14 (10.5) | 55* (41.3) |  |
|  |  | Skin | 133 | 3 (2.2) | 8 (6.01) | 1 (0.7) | 15 (11.3) | 24* (18.04) |  |
| Goats | Campo | Blood | 39 | 5 (12.8) | 3 (7.7) | 1 (2.6) | 9 (23.1) | 16* (41.02) | **30** (39.5)** |
|  |  | Skin | 39 | 6 (15.4) | 2 (5.1) | 0 (0) | 4 (10.2) | 9* (23.1) |  |
|  | Bipindi | Blood | 37 | 8 (21.6) | 0 (0) | 0 (0) | 1 (2.7) | 9 (24.3) |  |
|  |  | Skin | 37 | 2 (5.4) | 0 (0) | 0 (0) | 0 (0) | 2 (5.4) |  |
|  | Total | Blood | 76 | 13 (17.1) | 3 (3.9) | 1 (1.3) | 10 (13.1) | 25* (32.9) |  |
|  |  | Skin | 76 | 8 (10.5) | 2 (2.6) | 0 (0) | 4 (5.3) | 11* (14.5) |  |
| Sheep | Campo | Blood | 18 | 7 (38.9) | 1 (5.5) | 1 (5.5) | 0 (0) | 8* (44.4) | **18* (40)** |
|  |  | Skin | 18 | 1 (5.5) | 2 (11.1) | 0 (0) | 1 (5.5) | 4 (22.2) |  |
|  | Bipindi | Blood | 27 | 3 (7.4) | 0 (0) | 0 (0) | 2 (7.4) | 5 (18.5) |  |
|  |  | Skin | 27 | 0 (0) | 0 (0) | 0 (0) | 1 (3.7) | 1 (3.7) |  |
|  | Total | Blood | 45 | 10 (20) | 1 (2.2) | 1 (2.2) | 2 (4.4) | 13* (28.9) |  |
|  |  | Skin | 45 | 1 (2.2) | 2 (4.4) | 0 (0) | 2 (4.4) | 5 (11.1) |  |
| Dog | Campo | Blood | 21 | 3 (14.3) | 1 (4.8) | 1 (4.8) | 0 (0) | 4* (19.05) | **8* (38.1)** |
|  |  | Skin | 21 | 4 (19.05) | 1 (4.8) | 0 (0) | 0 (0) | 5 (23.8) |  |
|  | Total | Blood | 21 | 3 (14.3) | 1 (4.8) | 1 (4.8) | 0 (0) | 4* (19.05) |  |
|  |  | Skin | 21 | 4 (19.05) | 1 (4.8) | 0 (0) | 0 (0) | 5 (23.8) |  |
| Wild animals | Campo | Blood | 11 | 4 (36.4) | 2 (18.2) | 0 (0) | 3 (27.3) | 6* (54.5) | **9* (56.2)** |
|  |  | Skin | 10 | 0 (0) | 1 (10) | 0 (0) | 2 (20) | 2* (20) |  |
|  | Bipindi | Blood | 5 | 1 (20) | 1 (20) | 0 (0) | 0 (0) | 2 (40) |  |
|  |  | Skin | 5 | 0 (0) | 0 (0) | 0 (0) | 0 (0) | 0 (0) |  |
|  | Total | Blood | 16 | 5 (31.2) | 3 (18.7) | 0 (0) | 3 (18.7) | 8* (50) |  |
|  |  | Skin | 15 | 0 (0) | 1 (6.7) | 0 (0) | 2 (13.3) | 2* (13.3) |  |
| **Total** | Campo | Blood | 200 | 52 (26) | 15 (7.5) | 4 (2) | 24 (12) | 83* (41.5) | **137** (47.1)** |
|  |  | Skin | 199 | 13 (6.5) | 14 (7.03) | 1 (0.5) | 19 (9.5) | 41* (20.6) |  |
|  | Bipindi | Blood | 91 | 15 (16.5) | 2 (2.2) | 0 (0) | 5 (5.5) | 22 (24.2) |  |
|  |  | Skin | 91 | 3 (3.3) | 0 (0) | 0 (0) | 4 (4.4) | 6* (6.6) |  |
|  | **Total** | **Blood** | **291** | **67 (22.7)** | **17 (5.8)** | **4 (1.4)** | **29 (9.96)** | **105* (36.1)** |  |
|  |  | **Skin** | **290** | **16 (5.5)** | **14 (4.8)** | **1 (0.34)** | **23 (7.9)** | **47* (16.2)** |  |

NEA: Number of Examined Animals; *Trypanozoon*: *Trypanosoma brucei s.l;* TCF: *Trypanosoma Congolense* forest type; TCN: *Trypanosoma congolense* savannah type; TVW: *Trypanosoma vivax,* * mixed infections; ** both blood and skin trypanosomes. The proportion of infected animals was compared between animal species, trypanosomes species and between blood and skin sample.
